# Plasma secretory phospholipase A2-IIa as a potential biomarker for lung cancer in patients with solitary pulmonary nodules

**DOI:** 10.1186/1471-2407-11-513

**Published:** 2011-12-09

**Authors:** Elena Kupert, Marshall Anderson, Yin Liu, Paul Succop, Linda Levin, Jiang Wang, Kathryn Wikenheiser-brokamp, Pingping Chen, Susan M Pinney, Trudy Macdonald, Zhongyun Dong, Sandra Starnes, Shan Lu

**Affiliations:** 1 Department of Cancer and Cell Biology, University of Cincinnati College of Medicine, Cincinnati, OH 45237, USA; 2 Department of Pathology, University of Cincinnati College of Medicine, UC Metabolic Diseases Institute, 2120 E. Galbraith Road, Building A, Room 259, Cincinnati, OH 45237-0507, USA; 3 Department of Environmental Health, University of Cincinnati College of Medicine, Cincinnati, OH 45237, USA; 4 Department of Medicine, University of Cincinnati College of Medicine, Cincinnati, OH 45237, USA; 5 Department of Surgery, University of Cincinnati College of Medicine, Cincinnati, OH 45237, USA

## Abstract

**Background:**

Five-year survival for lung cancer has remained at 16% over last several decades largely due to the fact that over 50% of patients are diagnosed with locally-advanced or metastatic disease. Diagnosis at an earlier and potentially curable stage is crucial. Solitary pulmonary nodules (SPNs) are common, but the difficulty lies in the determination of which SPN is malignant. Currently, there is no convenient and reliable biomarker effective for early diagnosis. Secretory phospholipase A2-IIa (sPLA2-IIa) is secreted into the circulation by cancer cells and may allow for an early detection of lung cancer.

**Methods:**

Plasma samples from healthy donors, patients with only benign SPN, and patients with lung cancer were analyzed. Expression of sPLA2-IIa protein in lung cancer tissues was also determined.

**Results:**

We found that the levels of plasma sPLA2-IIa were significantly elevated in lung cancer patients. The receiver operating characteristic curve analysis, comparing lung cancer patients to patients with benign nodules, revealed an optimum cutoff value for plasma sPLA2-IIa of 2.4 ng/ml to predict an early stage cancer with 48% sensitivity and 86% specificity and up to 67% sensitivity for T2 stage lung cancer. Combined sPLA2-IIa, CEA, and Cyfra21.1 tests increased the sensitivity for lung cancer prediction. High level of plasma sPLA2-IIa was associated with a decreased overall cancer survival. sPLA2-IIa was overexpressed in almost all non-small cell lung cancer and in the majority of small cell lung cancer by immunohistochemistry analysis.

**Conclusion:**

Our finding strongly suggests that plasma sPLA2-IIa is a potential lung biomarker to distinguish benign nodules from lung cancer and to aid lung cancer diagnosis in patients with SPNs.

## Background

Lung cancer is the leading cause of cancer death worldwide with 159,390 deaths in the US in 2009. This represents 30% of total cancer deaths [1]. The 5-year survival rate is only 16%, which has not changed in three decades. One key to improving lung cancer survival is to diagnose it at an earlier stage, given that the 5-year survival rate with stage 1A non-small cell lung cancer (NSCLC) is as high as 73% [2]. Advanced imaging technology and widespread availability of low dose spiral computed tomography (LDCT) have led to a significant increase in detection of lung nodules [3,4]. The prevalence of solitary pulmonary nodules (SPNs), (by definition, a SPN is < 3 cm in diameter), is 10 ~ 20% nationwide and up to 61% in the Ohio valley [5]. The majority of these nodules are benign, even among patients at increased risk for lung cancer such as smokers. The difficulty lies in the determination of which SPN is malignant. An increase in the size of a SPN by repeated CT scans is currently one important parameter to predict the presence of malignancy [6,7]. SPNs can be challenging to manage and costly to monitor with radiation exposure of repeat CT scans and potential morbidity of biopsy or surgical resection of benign nodules. In North America, surgical resection of a lung nodule alone costs more than $20,000 [8]. Identification of malignant nodules is crucial because they represent a localized and potentially curable form of lung cancer. A few plasma biomarkers have been used to screen and diagnose cancers [9-11]. Currently, there is no single inexpensive, convenient, and reliable biomarker proven effective for the diagnosis of lung cancer [12].

Secretory phospholipase A2-IIa (sPLA2-IIa) is a phospholipid hydrolase enzyme that mediates the release of arachidonic acid (AA) and lysophosphatidylcholine, which are the precursors of eicosanoids and platelet-activating factor, respectively [13,14]. Eicosanoids are products of both sPLA2-IIa and cyclooxygenase-2 (Cox-2) and exert control over many physiologic processes, such as inflammation, immunity, and tumorigenesis. It was reported that elevated eicosanoids, such as prostaglandins, are involved in the pathogenesis of lung cancer [15]. sPLA2-IIa is a downstream effector of the NF-kB gene [16,17]. A few early studies have demonstrated that sPLA2-IIa is overexpressed in almost all human prostate cancer specimens and elevated levels of sPLA2-IIa are associated with tumor grade [18-20]. sPLA2-IIa remains elevated in androgen-independent prostate cancers failing hormonal therapy and functions as a growth factor to stimulate growth in prostate cancer cells [21].

We recently reported that elevated signaling of the HER/HER2-PI3K-Akt-NF-kB pathway contributes to sPLA2-IIa overexpression and secretion in prostate cancer cells [17,22,23]. We confirmed that sPLA2-IIa secreted by cancer cells into the circulation to a detecTable level in mice carrying xenograft human tumor. We are the first to demonstrate that there are high levels of plasma sPLA2-IIa in prostate cancer patients, which are associated significantly with high Gleason score and advanced disease stage. In the present study, we expanded our observation to lung cancer. We found that lung cancer tissues overexpress sPLA2-IIa and high levels of plasma sPLA2-IIa may serve as a plasma biomarker for lung cancer.

## Methods

### Patient population

The study was conducted in accordance with the Declaration of Helsinki and the local Institutional Review Board approved the study protocol. All the specimens for this study were archived biospecimens with de-identified patient information. As part of the University of Cincinnati Thoracic Tumor Registry, plasma samples were collected pre-operatively from patients with pulmonary nodules known or suspected to be lung cancer undergoing resection (Principal investigator: Dr. Sandra Starnes) [5]. Data on final pathology of the resected nodules was collected and defined as the benign nodule-lung cancer cohort (BNLCC). Plasma samples were also collected from lung cancer patients from the "Genetic Epidemiology of Lung Cancer" *cohort *(*GELCC*; PI: Dr. Marshall Anderson), a familial lung cancer cohort. Plasma samples from healthy donors were obtained from the Cincinnati Hoxworth Blood Center.

### Reagent

The mouse sPLA2-IIa antibody is a custom antibody developed in our lab. Human sPLA2-IIa antibody for Immunohistochemical (IHC) was from LifeSpan BioSciences (Seattle, WA).

### Enzyme-linked immunosorbent assay (ELISA)

sPLA2-IIa levels in plasma samples were determined by ELISA kit (Catalog No. 585000, Cayman Chemical Company). All plasma samples were diluted ten times for ELISA as described before [17]. The concentration of sPLA2-IIa in plasma was tested in duplicate and determined against a standard curve for each ELISA assay. ELISA kits for Carcinoembryonic Antigen (CEA) (EIA-1871) and Cytokeratin-19 fragment (CYFRA 21.1) (EIA-3943) were purchased from DRG International Inc., USA. Plasma samples were subjected to CEA and Cyfra21.1 ELISA and quantitated against a standard curve of each ELISA assay.

### Immunohistochemical (IHC) staining

Lung disease spectrum tissue array was obtained from Biomatrix.US (Rockville, MD). Immunohistochemistry (IHC) for the sPLA-IIa protein was performed as detailed in our previous studies [17,24]. Briefly, paraffin-embedded tissue sections were deparaffinized in xylene, rehydrated in graded alcohol, and transferred to PBS. The slides were treated with a citric acid-based antigen-retrieval buffer (DAKO Co., Carpinteria, CA), followed by 3% H_2_O_2 _in methanol, incubated in blocking buffer (5% BSA and 5% horse serum in PBS) and then in the blocking buffer containing the primary antibody. After washing, the slides were incubated with a biotinylated secondary antibody (BioGenex Laboratories, San Ramon, CA), followed by washing and incubation with the streptavidin-conjugated peroxidase (BioGenex). A positive reaction was visualized by incubating the slides with sTable diaminobenzidine and counterstaining with Gill's hematoxylin (BioGenex) and mounted with Universal Mount mounting medium (Fisher Scientific, Pittsburgh, PA). The intensity and extent of positive labeling for sPLA2-IIa in tissue arrays were assessed semiquantitatively and scored as 0 (no staining), 1+ (weak and focal staining in < 25% of tissue), 2+ (moderate intensity in 25-50% of tissue), 3+ (moderate intensity in > 50% of tissue), and 4+ (strong and diffused staining in > 50% of tissue).

### Statistical Analysis

The means and standard deviations were calculated with confirmed significant difference in plasma sPLA2-IIa level between lung cancer specimens and benign nodule specimens. Geometric means and standard deviations were also calculated with confirmed significant difference in plasma sPLA2-IIa level between lung cancer specimens and clinical control specimens. Unpaired t test with Welch correction was performed to evaluate the difference between sPLA2-IIa means of (i) 96 lung cancer specimens versus 29 benign SPN specimens from the BNLCC; (ii) 44 T1 stage lung cancer specimens versus 18 T2 stage lung cancer specimens from the BNLCC; (iii) 44 lung cancer specimens from the GELCC versus 29 benign SPN specimens from the BNLCC. Unpaired t test with Welch correction was also used to analyze overall cancer survival year associated with plasma sPLA2-IIa level from the BNLCC.

A parametric Receiver Operating Characteristic (ROC) analysis of plasma sPLA2-IIa values was performed to evaluate the ability of the levels of plasma sPLA2-IIa to distinguish 96 patients with lung cancer from 29 patients with benign SPNs from the BNLCC. 44 lung cancer specimens from the GELCC relative to 29 benign SPNs specimens from the BNLCC were also subjected to the ROC analysis. The optimum cutoff value of plasma sPLA2-IIa was determined by calculating the Youden Index [25], which separated the combined set of sPLA2-IIa values into two groups, such that the number of correctly classified specimens was maximized, and the associated sensitivity and specificity for predicting lung cancer versus non-malignant nodule were determined.

## Results

### The levels of plasma sPLA2-IIa are increased in lung cancer patients

**Figure 1:**
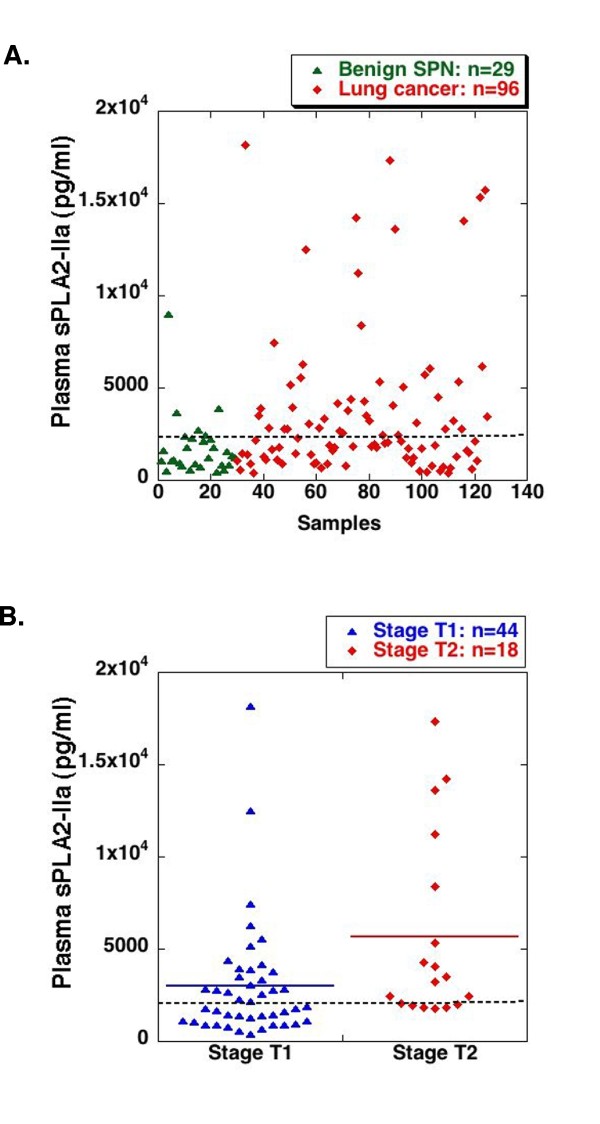
**High levels of plasma sPLA2-IIa are associated with lung cancer as compared with benign lung nodule**. (**a**) Plasma samples were diluted ten times and then subjected to ELISA analysis for sPLA2-IIa levels in duplicate of each sample. The average of the duplicate samples was calculated against to the standard curve in each experiment and presented as pg/ml sPLA2-IIa. (**b**) High levels of plasma sPLA2-IIa are significantly associated with T2 lung cancer relative to T1 stage lung cancer. The dotted line indicates the optimum cutoff value of 2.4 ng/ml

**Figure 2:**
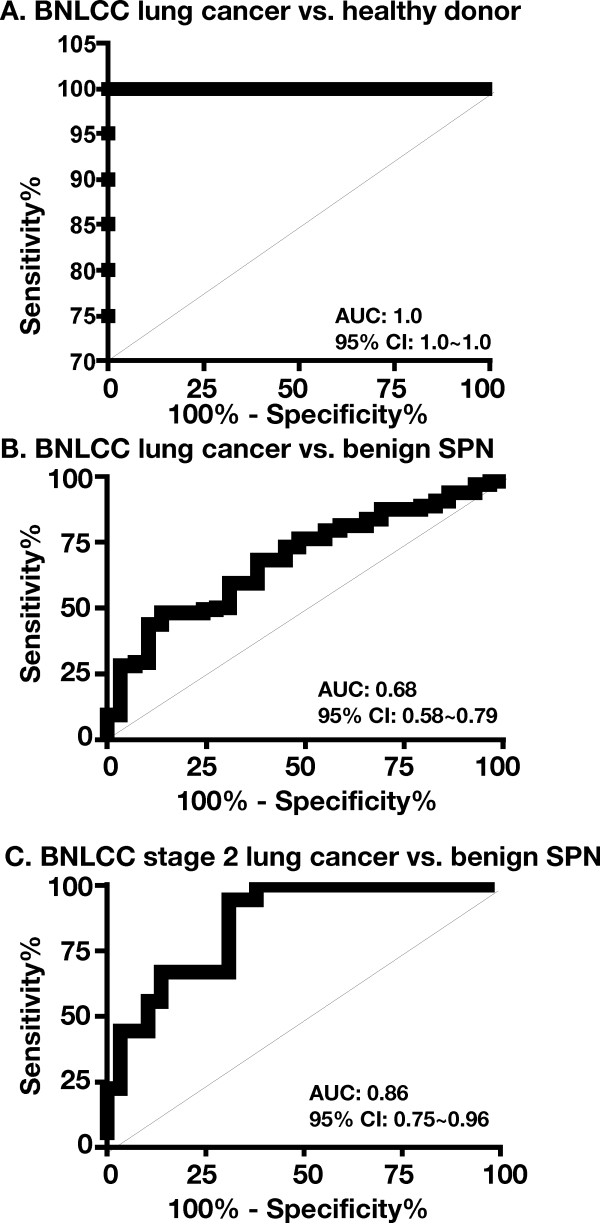
**Determination of plasma sPLA2-IIa to predict lung cancer by ROC analysis**. 96 Lung cancer specimens from the BNLCC relative to 20 healthy donors (**a**), 96 Lung cancer specimens relative to 29 benign SPN specimens from the BNLCC (**b**), and 18 T2 stage lung cancer specimens relative to 29 benign SPN specimens from the BNLCC were subjected to ROC analysis. Area under the ROC curve (AUC) and 95% confidence interval (95% CI) were determined.

The means and standard deviations of plasma sPLA2-IIa levels from 96 patients with lung cancer and 29 patients with benign lung nodules (SPNs) from the BNLCC were 3646 ± 407.3 and 1772 ± 306.8 pg/ml, respectively (Table 1 and 2, and see Additional file 1). Based on an unpaired t-test, the average plasma sPLA2-IIa level in lung cancer patients was significantly higher than that in the non-malignancy SPN controls (*P *= 0.004). The optimum cutoff value of plasma sPLA2-IIa by ROC analysis was 2.4 ng/ml, which resulted in 48% sensitivity and 86% specificity for predicting the presence of lung cancer (Figure 1a and Table 3). The area under the curve (AUC) was 0.68 (95% CI: 0.58 ~ 0.79) (Figure 2b). We also analyzed 44 lung cancer samples from the GELCC in which mean and standard deviation of plasma sPLA2-IIa were 3718 ± 407.8 (see Additional file 2). The average plasma sPLA2-IIa level in these lung cancer patients was also significantly higher than that in patients with benign SPNs from the BNLCC (*P *= 0.003). The optimum cutoff value of plasma sPLA2-IIa by ROC analysis was 2.4 ng/ml, which resulted in 64% sensitivity and 86% specificity for predicting lung cancer (AUC: 0.79; 95% CI: 0.69 ~ 0.90) (Table 3a). On the other hand, among 20 healthy donors, the levels of plasma sPLA2-IIa were undetecTable in 15 healthy donors, while those of the remaining 5 donors ranged up to 275 pg/ml (see Additional file 3). Age was not significantly associated with the level of plasma sPLA2-IIa in this healthy donor cohort (Table 1). ROC analysis of the plasma sPLA2-IIa levels in 96 lung cancer samples from the BNLCC and 20 healthy donor samples revealed an AUC as 1.0 (95% CI: 1.0 ~ 1.0) (Figure 2a).

**Table 1:** Characteristics of the patients

BNLCC-Lung cancer		96
M/F		37/59

Mean age year (range)		64 (41~88)

NSCLC		93

	Adenocarcinoma	54

	Squamous carcinoma	21

	Other NSCLC	18

SCLC		3

Stage		

	I	44

	II	18

	III+IV	4

Blooddraw before resection		96

Blooddraw after resection		0

		

**BNLCC-benign SPN**		29

M/F		15/14

Mean age year (range)		56 (31~81)

Blooddraw before resection		29

Blooddraw after resection		0

		

**GELCC-Lung cancer**		44

M/F		18/26

Mean age year (range)		61 (47~70)

		

**Healthy donor**		20

Mean age year (range)		49 (24~65)

**Table 2:** Plasma sPLA2-IIa levels and diagnosis in patients with benign SPNs from BNLCC

Sample	sPLA2-IIa (pg/ml)	Diagnosis
1	1039.2	0.9 cm nodule and heavy smoker

2	1589.2	1.3 × 1.4 × 1.1 cm subpleural nodule, necrotizing granuloma

3	480.83	Nodule 2 cm

4	**8980.8**	Necrotizing granulomatous

5	1030.8	Nonnecrotizing granulomas

6	1105.8	Abscess colonized with fungal hyphae

7	**3680.8**	Nodule with organizing pneumonia pattern of lung injury

8	922.5	Caseating granulomas with emphysematous changes

9	797.5	Nodules of caseating granulomas

10	2372.5	Necrotizing granuloma with fungal organisms

11	1789.2	Peribronchial tumorlet-2 mm and focal parenchymal fibrosis

12	580.83	Necrotizing granuloma with fungal organisms

13	2305.8	Necrotizing granuloma (2 × 1.7 × 1.3 cm)

14	872.5	Broncial and broncheolar ectasis with fibrosis and chronic inflammation c/w bronchiectasis

15	**2714.2**	Bronchiolitis obliterans organizing pneumonia

16	714.17	Focal fibrosis and chronic inflammation and reactive pneumocyte hyperplasia

17	2105.8	Caseating granulomas with fungal elements

18	**2422.17**	Myolipomatous polyp

19	1226.52	Active granulomatous inflammation and possible sarcoidosis

20	2217.83	Neurofibroma, 4.5 × 3.5 cm

21	1804.78	Active non-caseating granulomas

22	430.87	Necrotizing granuloma with fungal organisms

23	**3878.7**	Caseating necrotizing granulomas with fungal elements

24	835.22	Non-caseating granulomas with fungal elements

25	572.5	Necrotizing granulomatous inflammation with fungal organisms consistent with Aspergillus

26	1553.75	Necrotizing granulomas with fungal yeast forms consistent with Histoplasma

27	816.25	Organizing suppurative bronchopneumonia with pleural adhesions

28	1328.75	Presented with sTable lung nodule

29	1228.13	necrotizing granuloma and Langerhans' cell histiocytosis

*The data in bold is higher than the cutoff value of the blood test.

**Table 3:** High level of plasma sPLA2-IIa predicts lung cancer

*Table 3a: Determination of plasma sPLA2-IIa to predict lung cancer by ROC analysis*.					
**Plasma sPLA2-IIa**	**< 2.4 ng/ml**	**> 2.4 ng/ml**	**Total case#**	**%Senstivity**	**%Specificity**

**BNLCC lung cancer vs. benign SPN**					

Benign SPN	24	5	29		**86**

Lung cancer	50	46	96	**48**	

Stage 1 lung cancer	24	20	44	**45**	

Stage 2 lung cancer	6	12	18	**67**	

**GELCC lung cancer vs. benign SPN**					

Lung cancer	16	28	44	**64**	

					

*Table 3b: Combined sPLA2-IIa, CEA, and Cyfra21.1 blood tests increase the sentisitivity* *to predict lung cancer*.					

**sPLA2-IIa**	**< 2.4 ng/ml**	**> 2.4 ng/ml**			

**CEA**	**< 6.0 ng/ml**	**> 6.0 ng/ml**	**Total case#**	**%Senstivity**	**%Specificity**

**Cyfra21.1**	**< 3.3 ng/ml**	**> 3.3 ng/ml**			

**BNLCC lung cancer vs. benign SPN**					

Benign SPN	22	7	29		**76**

Lung cancer	36	60	96	**63**	

Stage 1 lung cancer	18	26	44	**59**	

Stage 2 lung cancer	5	13	18	**72**	

					

**CEA**	**< 6.0 ng/ml**	**> 6.0 ng/ml**	**Total case#**	**%Senstivity**	**%Specificity**

**BNLCC lung cancer vs. benign SPN**					

Benign SPN	28	1	29		**97**

Lung cancer	77	19	96	**20**	

					

**Cyfra21.1**	**< 3.3 ng/ml**	**> 3.3 ng/ml**	**Total case#**	**%Senstivity**	**%Specificity**

**BNLCC lung cancer vs. benign SPN**					

Benign SPN	27	2	29		**93**

Lung cancer	83	13	96	**14**	

Further analysis revealed that plasma sPLA2-IIa was significantly higher in T2 stage lung cancer (*n *= 18) relative to T1 stage lung cancer (*n *= 44) from the BNLCC (Two tailed t test: *P *= 0.005) (Figure 1b and see Additional file 1). The optimum cutoff value of plasma sPLA2-IIa by ROC analysis was 2.4 ng/ml, which resulted in 67% sensitivity for predicting T2 stage lung cancer (AUC: 0.86; 95% CI: 0.65 ~ 0.96), as compared with 45% sensitivity for predicting T1 stage lung cancer (Table 3a and Figure 2c). High levels of plasma sPLA2-IIa were also associated with a decreased overall survival (Unpaired t test with Welch correction: *P *= 0.0457) (see Additional file 1). The mean overall survival year for high sPLA2-IIa (> 2.4 ng/ml, *n *= 6) was 1.8 ± 1.3 years, while the mean overall survival year for low sPLA2-IIa (< 2.4 ng/ml, *n *= 11) was 3.3 ± 1.9 years.

Given the heterogeneous nature of cancer, the combined blood tests including a panel of biomarkers will increase the sensitivity for cancer prediction. Carcinoembryonic Antigen (CEA) and Cytokeratin-19 fragment (Cyfra 21.1) with the optimum cutoff value 6 ng/ml and 3.3 ng/ml, respectively, are two best biomarkers for predicting NSCLC currently under investigation [12,26,27]. Positive prediction for the presence of lung cancer by the combined blood tests was defined as one or more biomarkers higher than their cutoff values. We found that a combination of sPLA2-IIa (2.4 ng/ml cutoff value), Cyfra 21.1 (3.3 ng/ml cutoff value), and CEA (6 ng/ml cutoff value) tests increased the sensitivity for lung cancer prediction up to 62% from 48% by sPLA2-IIa test alone for 96 cancers relative to 29 patients with benign SPNs from the BNLCC (Table 3b and see Additional file 1 and 4). Furthermore, the combined tests increased the sensitivity for lung cancer prediction up to 59% and 72% from 45% and 67% by sPLA2-IIa test alone for 44 T1 stage cancers and 18 T2 stage cancers relative to 29 patients with benign SPNs, respectively. CEA and Cyfra 21.1 test alone had only 20% and 14% sensitivity, respectively (Table 3b).

Among the 5 patients with benign lung nodules and higher level of plasma sPLA2-IIa than the cutoff value 2.4 ng/ml, two patient suffered from localized pneumonia, one patient suffered from myolipomatous polyp, and the other two patients were diagnosed with necrotizing granulomas (Table 2). This finding indicated that active localized inflammation can occasionally lead to a moderate increased plasma sPLA2-IIa. Furthermore, our data demonstrated that there was an increased basal level of plasma sPLA2-IIa in patients with SPNs relative to those in healthy donors, which may result from SPNs and chronic inflammation.

Our data strongly suggest that plasma sPLA2-IIa can serve as a biomarker to predict more than 48% of T1 stage lung cancers and up to 67% of T2 stage lung cancer relative to patients with benign SPNs, although the plasma sPLA2-IIa is elevated occasionally in patients of benign SPNs. sPLA2-IIa is the best biomarker relative to Cyfra 21.1 and CEA, and the combined sPLA2-IIa, Cyfra 21.1 and CEA tests increase the sensitivity for lung cancer prediction relative to sPLA2-IIa test alone. In addition, plasma sPLA2-IIa may potentially serve as a poor prognosis biomarker for lung cancer.

### sPLA2-IIa is overexpressed in lung cancer specimens

**Figure 3:**
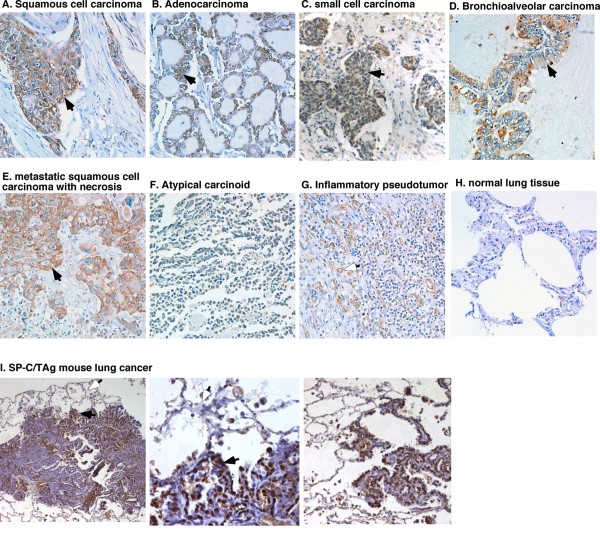
**IHC analysis of sPLA2-IIa expression in lung cancer specimens**. Brown staining indicates positivity for sPLA2-IIa. **a-e**. All primary and metastatic tumors indicate positive staining for sPLA2-IIa (solid arrow). **g**. Some endothelial cells in new blood vessels (open arrow) and macrophages show positive staining for sPLA2-IIa in inflammatory pseudo tumor. Atypical carcinoid (**f**) and normal lung (**h**) tissue are negative staining for sPLA2-IIa. (**i**) sPLA2-IIa overexpression was found in the spontaneous mouse lung cancer specimens of SP-C/TAg transgenic mice (solid arrow), but not in the adjacent normal type I and II epithelial cells (open arrow)

Among 100 core lung biopsies examined by IHC, sPLA2-IIa was overexpressed in 100% of squamous cell carcinoma (20 cores), adenocarcinoma (20 cores), and bronchioalveolar carcinoma (10 cores), in 70% of small cell carcinoma (10 cores), and in 90% of metastatic squamous cell carcinoma (10 cores) (Figure 3a-e), but not detected in atypical carcinoid (5 cores) (Figure 3f). sPLA2-IIa was not detected in inflammatory pseudo tumor (5 cores), tuberculosis (5 cores), and normal lung tissue (15 cores) (Figure 3g-h). Expression of sPLA2-IIa in infiltrating macrophages and endothelial cells of new blood vessels in inflammatory pseudo tumors (Figure 3g) implicated an underlying mechanism of localized inflammation as the cause of occasional elevated levels of plasma sPLA2-IIa in patients with benign lesions (Table 2). We also found that sPLA2-IIa was overexpressed in lung cancer tissue, but not the adjacent normal type I and II epithelial cells in the spontaneous mouse lung cancer from SP-C/TAg transgenic mice, in which the transgene SV40 early region (TAg) gene was driven by a 3.7 kb promoter of human surfactant protein C (SP-C) gene (Figure 3i) [28,29]. These data confirmed that sPLA1-IIa was overexpressed in lung cancer cells, but not in normal alveolar epithelial cells.

## Discussion

With increased sensitivity of imaging technology, such as low dose spiral computed tomography (LDCT), lung cancer can be detected at an earlier stage [2]. Recently, lung cancer screening with CT has been shown to detect lung tumors earlier and decrease lung cancer mortality [30]. One of the problems with the use of CT screening is the number of false positive with up to 25% of the subjects in the lung cancer screening trials having nodules, most of which are benign. Given the epidemic of histoplasmosis in the Ohio valley, we found that among 132 people enrolled in a pilot lung cancer screening project, 61% of subjects had nodules, most of which were benign [5]. If lung cancer screening with CT becomes standard, there will be a large number of nodules that will need to be evaluated. Monitoring these nodules usually requires repeated CT scans at 3, 6, 9, 12, 24 months based on the sizes of SPNs [6]. An increase in the size of a SPN is currently the parameter used to predict the presence of lung cancer. This repeated CT scan results in radiation exposure as well as anxiety. Patients may also undergo invasive procedures for diagnosis such as bronchoscopy, CT-guided fine needle aspiration, or thoracoscopic resection. It will become critical to have more effective ways to determine which nodules are most likely malignant.

We recently reported that there is an elevated level of plasma sPLA2-IIa in prostate cancer patients [17]. The current study showed that lung cancer tissues overexpress sPLA2-IIa and that patients with lung cancer have elevated levels of plasma sPLA2-IIa. The expression of the sPLA2-IIa gene is not tissue-specific or cancer-specific [17]. It was reported that the levels of plasma sPLA2-IIa are elevated with bacterial and viral infection or IL-2 infusion [31,32] and in coronary heart disease [33-35]. However, a recent clinical study including more than 500 patients showed that there is no significant alteration in plasma level of sPLA2-IIa protein among patients with coronary artery disease relative to healthy controls [34]. In consideration of these complications, we included some patients with inflammatory nodules of the lung as controls. We found an increased basal level of plasma sPLA2-IIa in these controls relative to those in healthy donors, which may be a result of chronic inflammation due to fungal infection and pneumonia. This active localized inflammation can occasionally lead to a moderate increased level of plasma sPLA2-IIa. Nevertheless, a significant high level of plasma sPLA2-IIa as a result of lung cancer can predict more than 48% of early stage lung cancers and up to 67% of T2 stage lung tumors with 86% specificity. sPLA2-IIa blood test has potential to help with the decision algorithm and determine the timing of subsequent CT scans and potential biopsy of concerning nodules.

One concern for using sPLA2-IIa as a lung cancer biomarker is that it lacks cancer specificity. This is one of the common features shared by all biomarkers currently in clinical use or under development and is simply due to the fact that genes contributing to cancer development overlap with those for other metabolic diseases, such as diabetes, obesity, and inflammation. In addition, the sensitivity of these plasma biomarkers is usually not very high, especially for early stages of cancer, which is partially due to the heterogeneous nature of cancer. Only a few plasma biomarkers are currently used to screen and diagnose cancers in clinical practice, including PSA, α-fetoprotein, CA19-9, CA125, and CEA [9-11]. None of these plasma biomarkers are cancer-specific [11]. For example, PSA is tissue-specific but not cancer-specific and has only 21% sensitivity with 4 ng/ml as the cutoff value [36]. Plasma PSA are elevated in benign diseases, such as in 30 ~ 50% BPH patients, which leads to low specificity [37]. On the other hand, many prostate cancers do not lead to a high PSA and fail to be detected in PSA screening, which also contributes to low sensitivity [37-41]. α-fetoprotein (AFP) for hepatocellular carcinoma has 65% sensitivity and 89% specificity, while the addition of VEGF and fucosidase (AFU) tests can increase the sensitivity up to 100% [42,43]. Furthermore, it is also noted that sensitivity of plasma biomarkers, such as CA125, is increased with advanced cancer stages, which is also consistent with our observation of plasma sPLA2-IIa for lung cancer [44].

A few serum biomarkers for lung cancer are currently under investigation, including Cyfra 21.1 and CEA for NSCLC and neuron-specific enolase (NSE) for SCLC [12,26,27]. Our data showed that none of these lung cancer biomarkers, based on the cutoff value reported, has high sensitivity to predict an early stage lung cancer [45]. However, the combined test of sPLA2-IIa, Cyfra 21.1 and CEA increases the power for lung cancer prediction relative to sPLA2-IIa test alone. We did not examine NSE, since there are only three SCLC specimens in the BNLCC cohort. Although Cyfra 21.1 is preferentially for squamous cell carcinoma, we did not find that Cyfra 21.1 shows a high sensitivity for squamous cell carcinoma. Given the heterogeneous nature of cancer and heterogeneous gene overexpression from one cancer to another, a combined blood test including a few plasma biomarkers is essential to increase the sensitivity to predict an early stage lung cancer.

In summary, we demonstrated that lung cancer tissues overexpress sPLA2-IIa and there is an elevated level of plasma sPLA2-IIa in lung cancer patients. Multiple lines of evidence support the notion that plasma sPLA2-IIa may represent a novel biomarker for lung cancer: 1) sPLA2-IIa is overexpressed in all squamous cell carcinoma, adenocarcinoma, and bronchioalveolar carcinoma examined and in majority of small cell carcinoma; 2) High levels of plasma sPLA2-IIa predict approximately 48% of early stage lung cancers and up to 67% of T2 stage lung cancers relative to patients with benign SPN; 3) High levels of plasma sPLA2-IIa are associated with advanced lung cancer stages and a decreased overall cancer survival; 4) the combined sPLA2-IIa, Cyfra 21.1 and CEA tests increase the power for lung cancer prediction relative to sPLA2-IIa test alone; 5) The combined lung cancer blood tests have potential to help with the decision algorithm of lung cancer diagnosis and reduce the number of CT scan and radiation exposure.

## Conclusions

This study uncovered that plasma sPLA2-IIa is a potential biomarker for lung cancer. sPLA2-IIa in combination with lung cancer biomarkers Cyfra 21.1 and CEA will help with the decision algorithm for diagnosis of an early stage lung cancer in patients with SPNs and poor prognosis. Application of this combined biomarker test will increase lung cancer survival, reduce painful and costly resection of SPN, and reduce radiation exposure by repeated CT exam for SPN patients.

## Abbreviations

sPLA2-IIa: Secretory phospholipase A2 group IIa; SPNs: Solitary pulmonary nodules; NSCLC: Non-small cell lung cancer; SCLC: Small cell lung cancer; Cyfra 21.1: Cytokeratin-19 fragment; CEA: Carcinoembryonic antigen; NSE: Neuron-specific enolase; ROC: Receiver operating characteristic; LDCT: Low dose spiral computed tomography

## Competing interests

The authors declare that they have no competing interests.

## Authors' contributions

EK, MA, KW, PC, SP, ZD, SS, and TM participated in patient specimens and data collection. JW and KW performed pathology analysis. PS and LL performed statistical analysis. YL carried out immunohistochemistry analysis and ELISA. MA, ZD, SS, and SL contributed to conception and design of study and interpretation of data. All authors read and approved the final manuscript.

## Pre-publication history

The pre-publication history for this paper can be accessed here:

http://www.biomedcentral.com/1471-2407/11/513/prepub

## Supplementary Material

Additional file 1 **Plasma sPLA2-IIa level in healthy donors**. Click here for file

Additional file 2 **The levels of plasma sPLA2-IIa, Cyfra21.1, and CEA in lung cancer patients from the BNLCC**. Click here for file

Additional file 3 **The levels of plasma sPLA2-IIa in lung cancer patients from the GELCC**. Click here for file

Additional file 4 **Plasma sPLA2-IIa, Cyfra21.1, and CEA levels and diagnosis in patients with benign SPNs from BNLCC**. Click here for file
