# Supplementary material for: Plasma secretory phospholipase A2-IIa as a potential biomarker for lung cancer in patients with solitary pulmonary nodules
Source: BMC Cancer. 2011 Dec 9;11:513. doi: 10.1186/1471-2407-11-513 (PMC3250967; doi:10.1186/1471-2407-11-513)
Supplement: Additional file 1 — Plasma sPLA2-IIa level in healthy donors. [file 1471-2407-11-513-S1.PDF]

Additional file 1: The levels of plasma sPLA2-IIa, Cyfra21.1, and CEA in lung cancer patients from the BNLCC

Sample	Stage	Diagnosis	Age	Sex	sPLA2-IIa (pg/ml)	Cyfra21.1 (ng/ml)	CEA (ng/ml)	Survival year
1	pT1	Squamous cell carcinoma	65	F	2151.43	0	0	
2	pT1	Adenocarcinoma	60	F	**18189**	0	0	
3	pT1	Adenocarcinoma	81	M	415.71	1.63	0	
4	pT1	Squamous cell carcinoma	63	M	564.17	0	0.840	4
5	pT1	Squamous cell carcinoma	75	F	1422.5	0	1.690	4
6	pT1	Squamous cell carcinoma	81	F	883.04	0	2.535	
7	pT1	Adenocarcinoma	61	F	1080.8	0	**9.249**	
8	pT1	Adenocarcinoma	69	M	1414.2	0	**13.286**	
9	pT1	Adenocarcinoma	79	F	**3505.88**	0	2.886	
10	pT1A	Squamous cell carcinoma	52	M	1130.8	0	0	
11	pT1A	Adenocarcinoma	76	F	1139.57	0	0	
12	pT1A	Adenocarcinoma	79	F	1661.3	0	0	
13	pT1A	Adenocarcinoma	68	M	1753.13	0	0	
14	pT1A	Adenocarcinoma	63	F	**2753.13**	0	0	
15	pT1A	Adenocarcinoma	43	F	**2814.2**	0	0	4
16	pT1A	Adenocarcinoma	44	F	**3914.2**	0	0	
17	pT1A	Adenocarcinoma	45	F	**3934.38**	0	0	
18	pT1A	Adenocarcinoma	51	M	884.38	2.88	0	
19	pT1A	Squamous cell carcinoma	66	M	**5171.88**	**8.98**	0	
20	pT1A	Adenocarcinoma	66	M	**7439.57**	**18.7**	0.469	
21	pT1A	Adenocarcinoma	71	F	1264.2	1.16	1.080	
22	pT1A	Adenocarcinoma	70	F	**2784.38**	**8.45**	**29.353**	
23	pT1a	Adenocarcinoma	48	F	1394.11	0	0	
24	pT1a	Adenocarcinoma	70	M	1452.94	0	0	
25	pT1a	Adenocarcinoma	62	F	**3070.58**	0	0	
26	pT1a	Squamous cell carcinoma	65	F	**5547.051**	0	0	
27	pT1a	Adenocarcinoma	64	F	**6288.23**	0	0	
28	pT1a	Squamous cell carcinoma	76	F	2300	**3.33**	0	
29	pT1a	Adenocarcinoma	76	F	**12482.35**	0	0	
30	pT1b	Carcinoid tumor	57	F	696.09	0	0	
31	pT1B	Adenocarcinoma	65	F	917.83	0	0	
32	pT1B	Adenocarcinoma	62	F	1604.78	0	0	
33	pT1B	Adenocarcinoma	68	F	**2678.7**	0	0	
34	pT1B	Adenocarcinoma	86	F	**3759.38**	0	0	
35	pT1B	Adenocarcinoma	65	M	**2530.87**	3.25	0	
36	pT1B	Carcinoma, spindled cell type	68	M	914.17	0	0.140	
37	pT1B	Adenocarcinoma	63	M	**4384.38**	0	0.249	
38	pT1B	Adenocarcinoma	64	F	**2830.8**	0	0.563	
39	pT1B	Adenocarcinoma	62	F	1783.04	**17.93**	3.380	
40	pT1B	Adenocarcinoma	81	F	**4170**	0	4.930	
41	pT1B	Squamous cell carcinoma	62	M	**3348.26**	1.35	5.023	1
42	pT1B	Adenocarcinoma	54	F	1874.35	0.53	5.446	2
43	pT1B	Adenocarcinoma	62	F	939.17	0	**12.911**	
44	pT1B	Adenocarcinoma	48	F	804.78	0	**14.836**	
45	pT2	Neuroendocrine carcinoma	88	M	1822.5	0	0	
46	pT2	Small cell carcinoma	65	F	**3251.43**	0	0	
47	pT2	NSCLC	55	M	**3501.43**	0	0	
48	pT2	NSCLC	56	M	**14223**	0	0	3
49	pT2	Adenosquamous Carcinoma	76	F	**11239.57**	0	0.235	
50	pT2	Squamous cell carcinoma	54	M	**8385**	0	1.315	
51	pT2	Squamous cell carcinoma	67	F	**4266.25**	0	2.160	
52	pT2A	adenocarcinoma	65	F	1828.13	0	**8.408**	
53	pT2a	adenocarcinoma	60	F	1782.35	0	0	
54	pT2a	NSCLC	68	F	**5358.82**	0	0.796	
55	pT2a	Adenocarcinoma	79	M	1941.17	0	0	
56	pT2B	Adenocarcinoma	64	F	**4078.13**	0	0	
57	pT2B	Squamous cell carcinoma	43	M	**17309.38**	**7.67**	0	
58	pT2B	NSCLC	77	F	2048.26	0	1.690	
59	pT2B	Squamous cell carcinoma	66	M	2026.52	0	4.225	5
60	pT2B	Squamous cell carcinoma	74	F	**2430.8**	0	**7.277**	
61	pT2B	adenocarcinoma	60	F	**13603.13**	**16.71**	**102.413**	
62	pT2b	adenocarcinoma	76	F	**2458.82**	0	0	
63	pT3	Squamous cell carcinoma	63	F	2089.2	0.66	0	1
64	pT3	Squamous cell carcinoma	69	F	**5039.2**	**4.33**	0	
65	pT3B	NSCLC	56	M	1209.13	0	0	2
66	pT4	Squamous cell carcinoma	57	M	1717.83	0	**21.221**	
67		Squamous cell carcinoma	67	M	397.5	0	0	2
68		Adenocarcinoma	62	M	505.83	0	0	
69		Adenocarcinoma	76	F	622.5	0	0	
70		Adenocarcinoma	63	M	685	0	0	
71		Small cell carcinoma	60	F	1044.29	0	0	
72		Adenocarcinoma	44	F	1239.2	0	0	3
73		Squamous cell carcinoma	63	M	1278.75	0	0	
74		Adenocarcinoma	58	F	2087.14	0	0	1
75		Squamous cell carcinoma	72	M	**2803.75**	0	0	1

76		Neuroendocrine w/brain mets	75	F	**3465.71**	0	0	
77		NSCLC	41	F	**4510**	0	0	
78		NSCLC	58	M	**14066.25**	0	0	1
79		Squamous cell carcinoma	87	M	**15351.43**	0	0	
80		Adenocarcinoma	73	M	**5735.22**	**3.6**	0	1
81		NSCLC	53	F	**5310**	**33.3**	0	
82		Adenocacinoma	52	F	1635	0	0.423	5
83		NSCLC	73	M	447.5	0	1.174	
84		Adenocarcinoma	66	F	**3130.8**	0	3.474	
85		NSCLC	52	M	**2760**	0	4.366	
86		Adenocarcinoma	62	M	1897.5	0	5.399	
87		NSCLC	67	M	1478.75	0	**7.960**	
88		Bronchial cancer	58	F	1722.5	0	**9.296**	
89		NSCLC	84	F	735	**3.44**	**13.380**	7
90		Adenocarcinoma	67	M	**3235**	0	**15.352**	
91		Adenocarcinoma	55	F	491.25	0	**29.014**	
92		Small cell carcinoma	58	F	**6187.14**	0	**58.209**	
93		Adenocarcinoma	45	M	772.5	**4.96**	**68.451**	
94		Adenocarcinoma	70	M	972.5	0	**70.939**	
95		Adenocarcinoma	45	F	**15715.71**	0	**71.866**	
96		NSCLC	62	F	**6060**	**175.56**	**201.268**	

*The data in bold is higher than the cutoff value of plasma sPLA2-IIa, Cyfra21.1, and CEA.
