# Supplementary material for: Plasma secretory phospholipase A2-IIa as a potential biomarker for lung cancer in patients with solitary pulmonary nodules
Source: BMC Cancer. 2011 Dec 9;11:513. doi: 10.1186/1471-2407-11-513 (PMC3250967; doi:10.1186/1471-2407-11-513)
Supplement: Additional file 2 — The levels of plasma sPLA2-IIa, Cyfra21.1, and CEA in lung cancer patients from the BNLCC. [file 1471-2407-11-513-S2.PDF]

Additional file 2: Plasma sPLA2-IIa level in healthy donors

Plasma samples	sPLA2-IIa (pg/ml)	Age
Healthy 1	0.00	53
Healthy 2	115.91	34
Healthy 3	0.00	48
Healthy 4	111.36	62
Healthy 5	145.45	60
Healthy 6	0.00	55
Healthy 7	0.00	46
Healthy 8	0.00	29
Healthy 9	0.00	64
Healthy 10	0.00	52
Healthy 11	217.73	64
Healthy 12	275.45	65
Healthy 13	0.00	61
Healthy 14	0.00	24
Healthy 15	0.00	39
Healthy 16	0.00	43
Healthy 17	0.00	55
Healthy 18	0.00	39
Healthy 19	0.00	48
Healthy 20	0.00	47
