# Supplementary material for: Plasma secretory phospholipase A2-IIa as a potential biomarker for lung cancer in patients with solitary pulmonary nodules
Source: BMC Cancer. 2011 Dec 9;11:513. doi: 10.1186/1471-2407-11-513 (PMC3250967; doi:10.1186/1471-2407-11-513)
Supplement: Additional file 3 — The levels of plasma sPLA2-IIa in lung cancer patients from the GELCC. [file 1471-2407-11-513-S3.PDF]

Additional file 3: The levels of plasma sPLA2-IIa in lung cancer patients from the GELCC

Sample	Age	Sex	Diagnosis	Stage	sPLA2-IIa pg/ml
1	47	M	lung cancer	N/D	**4133.08**
2	48	M	lung cancer	N/D	**4058.82**
3	51	M	SCLC	N/D	**3672.50**
4	53	M	adenocarcinoma	N/D	**3058.82**
5	54	F	adenocarcinoma	N/D	**5372.86**
6	54	M	squamous cell carcinoma	pT2N2	**3479.23**
7	55	M	squamous cell carcinoma	N/D	1015.71
8	55	F	adenocarcinoma	N/D	**5123.52**
9	56	F	adenocarcinoma	pT1x	1008.57
10	56	F	bronchioalveolar carcinoma	pT1	1864.7
11	57	F	NSCLC	pT4	**7072.35**
12	57	M	NSCLC	N/D	**6035.29**
13	57	F	adenocarcinoma	N/D	1952.94
14	57	F	adenocarcinoma	pT2	**4794.11**
15	57	F	adenocarcinoma	N/D	**3505.88**
16	58	F	NSCLC	pT3	**10501.43**
17	58	F	adenocarcinoma	pT1	1141.17
18	59	M	squamous cell	N/D	**2478.24**
19	61	M	squamous cell	N/D	**2917.69**
20	62	F	NSCLC	pT4	**13801.43**
21	62	F	adenocarcinoma	pT1	2065.71
22	62	M	NSCLC	N/D	2230.00
23	62	F	SCLC	N/D	1336.25
24	63	F	large cell carcinoma	N/D	1308.57
25	63	F	NSCLC	pT4	**4551.43**
26	63	M	adenocarcinoma	pT2	930.00
27	63	F	adenocarcinoma	pT3	1231.18
28	63	F	NSCLC	N/D	**3972.50**
29	64	M	lung cancer	N/D	**2586.25**
30	64	M	adenocarcinoma	N/D	**3456.15**
31	64	F	adenocarcinoma	pT2	2235.29
32	65	F	adenocarcinoma	N/D	**3261.25**
33	65	F	adenocarcinoma	pT1	**2761.25**
34	66	F	lung cancer	pT2	858.57
35	66	M	lung cancer	N/D	**5530.00**
36	67	F	adenocarcinoma	pT2	**5248.75**
37	67	M	squamous cell carcinoma	pT3	**2602.31**
38	67	M	lung cancer	N/D	**3235.29**
39	68	F	large cell carcinoma	N/D	1779.23
40	68	F	SCLC	N/D	**8035.00**
41	68	M	squamous cell carcinoma	N/D	1452.94
42	68	F	adenocarcinoma	N/D	**5782.35**
43	69	M	adenocarcinoma	pT1	1394.62
44	70	F	NSCLC	N/D	**8758.82**

*The data in bold is higher than the cutoff value of the blood test.
