# Supplementary material for: Plasma secretory phospholipase A2-IIa as a potential biomarker for lung cancer in patients with solitary pulmonary nodules
Source: BMC Cancer. 2011 Dec 9;11:513. doi: 10.1186/1471-2407-11-513 (PMC3250967; doi:10.1186/1471-2407-11-513)
Supplement: Additional file 4 — Plasma sPLA2-IIa, Cyfra21.1, and CEA levels and diagnosis in patients with benign SPNs from BNLCC. [file 1471-2407-11-513-S4.PDF]

Additional file 4: Plasma sPLA2-IIa, Cyfra21.1, and CEA levels and diagnosis in patients with benign SPNs from BNLCC

Sample	Age	Sex	sPLA2-IIa (pg/ml)	Cyfra21.1(ng/ml)	CEA (ng/ml)	Diagnosis
1	57	M	1039.2	0	0.000	0.9cm nodule and heavy smoker
2	40	F	1589.2	0	2.207	1.3 x 1.4 x 1.1 cm subpleural nodule, necrotizing granuloma
3	55	M	480.83	0	0.000	Nodule 2 cm
4	58	F	**8980.8**	2.3	0.000	Necrotizing granulomatous
5	47	F	1030.8	0	1.127	Nonnecrotizing granulomas
6	71	F	1105.8	0	0.986	Abscess colonized with fungal hyphae
7	44	F	**3680.8**	0	0.000	Nodule with organizing pneumonia pattern of lung injury
8	73	M	922.5	0	0.798	Caseating granulomas with emphysematous changes
9	57	M	797.5	0	0.094	Nodules of caseating granulomas
10	48	F	2372.5	0	3.239	Necrotizing granuloma with fungal organisms
11	72	M	1789.2	0	0.000	Peribronchial tumorlet-2mm and focal parenchymal fibrosis
12	36	F	580.83	0	0.000	Necrotizing granuloma with fungal organisms
13	53	M	2305.8	0	**12.582**	Necrotizing granuloma (2 x 1.7 x 1.3 cm)
14	49	F	872.5	0	1.784	Broncial and broncheolar ectasis with fibrosis and chronic inflammation c/w bronchiectasis
15	52	M	**2714.2**	0	1.737	Bronchiolitis obliterans organizing pneumonia
16	48	M	714.17	0	0.000	Focal fibrosis and chronic inflammation and reactive pneumocyte hyperplasia
17	52	M	2105.8	0	0.000	Caseating granulomas with fungal elements
18	48	M	**2422.17**	**14.9**	0.000	Myolipomatous polyp
19	64	F	1226.52	0	0.000	Active granulomatous inflammation and possible sarcoidosis
20	31	F	2217.83	0	0.000	Neurofibroma, 4.5 x 3.5 cm
21	72	F	1804.78	0.22	0.000	Active non-caseating granulomas
22	46	M	430.87	0	0.000	Necrotizing granuloma with fungal organisms
23	72	F	**3878.7**	0	0.000	Caseating necrotizing granulomas with fungal elements
24	67	F	835.22	0	0.000	Non-caseating granulomas with fungal elements
25	65	M	572.5	0	0.000	Necrotizing granulomatous inflammation with fungal organisms consistent with Aspergillus
26	81	M	1553.75	0	0.000	Necrotizing granulomas with fungal yeast forms consistent with Histoplasma
27	46	M	816.25	0	0.100	Organizing suppurative bronchopneumonia with pleural adhesions
28	72	M	1328.75	**8.32**	0.000	Presented with lung nodule. Three years post blood draw, the nodule remained stable in size
29	53	F	1228.13	0	1.194	necrotizing granuloma and Langerhans' cell histiocytosis

*The data in bold is higher than the cutoff value of the blood test.
